# Supplementary material for: Post-Neoadjuvant Surveillance and Surgery as Needed Compared with Post-Neoadjuvant Surgery on Principle in Multimodal Treatment for Esophageal Cancer: A Scoping Review
Source: Cancers (Basel). 2021 Jan 23;13(3):429. doi: 10.3390/cancers13030429 (PMC7865772; doi:10.3390/cancers13030429)
Supplement: Supplementary file 1 [file cancers-13-00429-s001.pdf]

Supplementary Materials: Post-Neoadjuvant Surveillance and Surgery as Needed Compared with Post-Neoadjuvant Surgery on Principle in Multimodal Treatment for Esophageal Cancer: A Scoping Review

Julian Hipp, Blin Nagavci, Claudia Schmoor, Joerg Meerpohl, Jens Hoeppner and Christine Schmucker

Search Strategies

Table S1. Search strategy for Medline (Ovid).

#	Searches
1	((esophag* or oesophag*) adj5 (cancer* or neoplas* or carcino* or tumor* or tumour* or malign* or adenocarcin* or adeno-carcin*)).ti,ab,kf.
2	esophageal neoplasms/ or esophageal squamous cell carcinoma/
3	1 or 2
4	(chemoradi* or radiochemo* or chemo-radi* or radio-chemo* or chemotherap* or Radiation or radiotherap*).ti,ab,kf.
5	exp Chemoradiotherapy/ or (Chemotherapy, Adjuvant/ and Radiotherapy,Adjuvant/)
6	4 or 5
7	(((watch* or see) adj3 wait*) or (active* adj3 surveil*) or ((selective* or needed or necessar* or unnecessar* or declin* or avoid* or on-demand) adj6 (resect* or surg* or esophagectom* or oesophagectom*)) or (chemoradiation alone or chemoradiation only or chemo-radiation alone or chemo-radiation only)).ti,ab,kf.
8	Watchful Waiting/
9	7 or 8
10	(surg* or standard treatment or standard therapy or standard surgical resection or tri-modal* or trimodal* or esophagectom* or oesophagectom*).ti,ab,kf.
11	exp Esophagectomy/
12	10 or 11
13	3 and 6 and 9 and 12
14	exp animals/ not exp humans/
15	editorial/ or letter/ or Congress/
16	13 not 14
17	16 not 15
18	limit 17 to (english or german)
19	randomized controlled trial.pt.
20	controlled clinical trial.pt.
21	randomized.ab.
22	placebo.ab.
23	drug therapy.fs.
24	randomly.ab.
25	trial.ab.
26	groups.ab.
27	19 or 20 or 21 or 22 or 23 or 24 or 25 or 26
28	3 and 9 and 27

29	28 not 14
30	28 not 15
31	limit 30 to (english or german)
32	18 or 31

Table S2. Search strategy for Cochrane Library.

ID	Search
#1	((esophag* OR oesophag*) NEAR/6 (Cancer* OR neoplas* OR carcino* OR adenocarcino* OR Tumor* OR tumour* OR malign*)):ti,ab,kw (Word variations have been searched)
#2	MeSH descriptor: [Esophageal Neoplasms] explode all trees
#3	#1 or #2
#4	((watch* OR see) NEAR/3 wait*):ti,ab,kw (Word variations have been searched)
#5	("chemoradiation alone" or "chemoradiation only" or "chemotherapy only" or "chemotherapy alone"):ti,ab,kw
#6	MeSH descriptor: [Watchful Waiting] explode all trees
#7	((selective* OR needed OR necessar* OR unnecessar* OR declin* OR avoid* OR on-demand) NEAR/6 (resect* OR surg* OR resect* OR esophagectom* OR oesophagectom*)):ti,ab,kw (Word variations have been searched)
#8	#4 or #5 or #6 or #7
#9	MeSH descriptor: [Chemoradiotherapy] explode all trees
#10	MeSH descriptor: [Radiotherapy] explode all trees
#11	(chemoradi* or radiochemo* or chemotherap* or radiation or radiotherap*):ti,ab,kw
#12	#9 or #10 #or11
#13	(surg* or "standard Treatment" or "standard therapy" or tri-modal* or trimodal* or esophagectom* or oesophagectom* or "standard surgical resection"):ti,ab,kw (Word variations have been searched)
#14	MeSH descriptor: [General Surgery] explode all trees
#15	MeSH descriptor: [Esophagectomy] explode all trees
#16	#13 or #14 or #15
#17	#3 and #8 and #16
#18	#3 and #8 and #12 and #16

Table s3. Search strategy for Web of Science.

# 6	#4 AND #3 AND #2 AND #1<br>**Refined by:** [excluding] **DOCUMENT TYPES:** (MEETING ABSTRACT)<br>*Indexes=SCI-EXPANDED Timespan=All years*
# 5	#4 AND #3 AND #2 AND #1<br>*Indexes=SCI-EXPANDED Timespan=All years*
# 4	TS=(surg* or standard treatment or standard therapy or standard surgical resection or tri-modal* or trimodal* or esophagectom* or oesophagectom*)<br>*Indexes=SCI-EXPANDED Timespan=All years*
# 3	TS=(((watch* or see) near/3 wait*) or (active* near/3 surveil*) or ((selective* or needed or necessar* or unnecessar* or declin* or avoid* or "on-demand") near/6 (resect* or surg* or esophagectom* or oesophagectom*)))<br>*Indexes=SCI-EXPANDED Timespan=All years*
# 2	TS= (chemoradi* or radiochemo* or chemotherap* or radiation or radiotherap*)<br>*Indexes=SCI-EXPANDED Timespan=All years*
# 1	TS=((esophag* OR oesophag*) NEAR/5 (cancer* OR neoplas* OR carcino* OR adenocarcino* OR tumor* OR tumour* OR malign*))<br>*Indexes=SCI-EXPANDED Timespan=All years*

Table s4. Search strategy for ScienceDirect [Elsevier].

Title, abstract, keywords: ("watchful waiting" OR "active surveillance" OR "wait-and see") AND Find articles with these terms (oesophageal OR esophageal)
Limits:<br>Article types: Review articles, Research articles
